# Method for Using Functional Near-Infrared Spectroscopy (fNIRS) to Explore Music-Induced Brain Activation in Orchestral Musicians in Concert

**DOI:** 10.3390/s25061807

**Published:** 2025-03-14

**Authors:** Steffen Maude Fagerland, Andreas Løve, Tord K. Helliesen, Ørjan Grøttem Martinsen, Mona-Elisabeth Revheim, Tor Endestad

**Affiliations:** 1 The Intervention Centre, Oslo University Hospital, 0372 Oslo, Norway; monar@ous-hf.no; 2 RITMO Centre for Interdisciplinary Studies in Rhythm, Time, and Motion, Department of Psychology, University of Oslo, 0313 Oslo, Norway; andreas.love@psykologi.uio.no (A.L.); tor.endestad@psykologi.uio.no (T.E.); 3 Department of Psychology, University of Oslo, 0313 Oslo, Norway; tordkh@uio.no; 4 Department of Physics, University of Oslo, 0313 Oslo, Norway; o.g.martinsen@fys.uio.no; 5 Department of Clinical and Biomedical Engineering, Oslo University Hospital, 0372 Oslo, Norway; 6 Department of Neuropsychology, Helgeland Hospital, 8657 Mosjøen, Norway

**Keywords:** fNIRS, brain imaging, hyperscanning, symphony orchestra, real-time 3D visualization

## Abstract

The act of performing music may induce a specific state of mind, musicians potentially becoming immersed and detached from the rest of the world. May this be measured? Does this state of mind change based on repetition? In collaboration with Stavanger Symphony Orchestra (SSO), we developed protocols to investigate ongoing changes in the brain activation of a first violinist and a second violinist in real time during seven sequential, public concerts using functional near-infrared spectroscopy (fNIRS). Using wireless fNIRS systems (Brite MKII) from Artinis, we measured ongoing hemodynamic changes and projected the brain activation to the audience through the software OxySoft 3.5.15.2. We subsequently developed protocols for further analyses through the Matlab toolboxes Brainstorm and Homer2/Homer3. Our developed protocols demonstrate how one may use “functional dissection” to imply how the state of mind of musicians may alter while performing their art. We focused on a subset of cortical regions in the right hemisphere, but the current study demonstrates how fNIRS may be used to shed light on brain dynamics related to producing art in ecological and natural contexts on a general level, neither restricted to the use of musical instrument nor art form.

## 1. Introduction

Music has a strong effect on the brain and may influence the perception and regulation of mood, behavior, cognitive factors, and movement; music may induce both simple and full-body motion [1,2]. How can the experience of performing with a symphony orchestra be described? The term musicking encapsulates both the creation and perception of music, encompassing both active and passive forms of engagement with music [3].

The benefits of music on physical health and well-being are increasingly being recognized [4]. Brain imaging and neurological investigations have traditionally implied that the experiments and mechanisms explored must be restricted to highly synthetic contexts, reducing the generalizability of the results to what may be learned about how these brain mechanisms relate to similar experiences in natural contexts [5].

In this study, we aimed to reduce this knowledge gap by applying functional near-infrared spectroscopy (fNIRS), recognized for its ecological validity ([6,7]), to investigate how the experience of two violinists performing with a symphony orchestra changes, in real time, and to develop methods for subsequent analyses after the concerts had concluded. We set out to investigate four questions:

1. How will an fNIRS hemodynamic 3D model representation of the experience of performing the violin in a symphony orchestra change in real time?
2. Will a method for studying/averaging the hemodynamic changes in recordings lasting the duration of each piece show systematic changes related to each performer?
3. Will a method for estimating the sources responsible for the hemodynamic changes show alterations in the most active brain regions throughout the seven concerts?
4. Regions of the brain with characteristic hemodynamic changes should alter with experience in line with the Predictive Coding of Music model. Will this technological setup corroborate such a statement?

**Figure 1:**
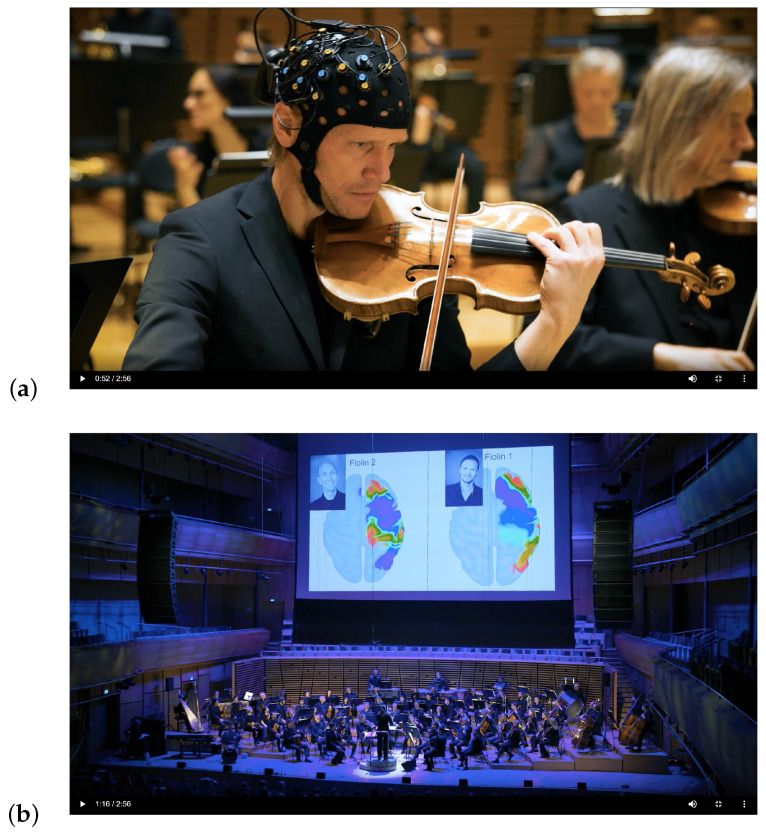
Overview of the project; (**a**) fNIRS setup of the 1st violinist; (**b**) real-time presentation of brain activation of the two violinists to the audience.

These questions set the stage for our collaboration with the Stavanger Symphony Orchestra (SSO) through the seven concerts (Figure 1).

### Measuring Music-Induced Brain Activation

Music influences us in a multitude of ways, igniting pleasurable and affective experiences and moving us emotionally and physically [8]. However, describing the experience of music is challenging. The experience of listening to music has been reviewed in phenomenological terms ([9]), in terms of its psychological functions ([10]), and regarding its biological impact ([11]). Performing music has similarly been assessed for how it is experienced as meaningful ([12]), the flow state that may be experienced ([13]), and the sensors that may be used to quantify the experience ([14]). How music is experienced is also inherently subjective [15].

Learning to play music may shape both brain structure and function [8] and enhance the development of syntax processing in children ([16]), and listening to familiar music may induce neuroplastic mechanisms that, for example, could help people with Alzheimer’s disease [17]. Music processing in the brain has traditionally been investigated as an auditory phenomenon through passive listening experiments ([8]), but parallels have been drawn to cognitive domains alongside language, and a model has been developed for the neural chronometry of the aesthetic experience of music [18]. A recent review highlighted how the act of performing music may impact emotion regulation [4].

In neuroscience, many technological instruments have been used to gain insight into the brain, all with their inherent strengths and limitations. It has consistently been shown across studies that music may evoke changes in the core structures of the brain underlying emotion [19]. The non-invasive method of electroencephalography (EEG), due to its safety, high resolution, and sensitivity to changes in the brain, has garnered significant interest within neuroscience [20]. However, functional magnetic resonance imaging (fMRI) has transformed the field of neuroimaging by imaging the brain through metabolic functions [21]. fMRI may investigate brain activity both during tasks and while resting ([22,23]) and has revealed, for example, differences in spontaneous activity patterns in children with early Tourette’s syndrome ([24]), differences in connectivity patterns for bipolar disorder ([25]), how medication may affect motivation ([26]), and how the brain reacts to mindfulness [27].

Due to the strong effect that music may have on the brain, it has also been used for therapeutic purposes. A recent review highlighted the effects of music therapy in reducing anxiety and depression in breast cancer patients ([28]). An RCT of a single session of on-site music therapy on anxiety and vital parameters for hospitalized COVID-19 patients showed an effect ([29]), and a meta-analysis/review indicated that the systematic use of music may enhance recovery in patients after stroke-induced motor dysfunction [30]. EEG and fMRI investigations have been used to explore the neural basis of music perception ([31]). fMRI studies have linked listening to music to core structures involved in reward pathways ([32]), and a review on music therapy for reducing cognitive impairment (through EEG and fMRI) highlighted potential effects and suggestions to maximize the impact of music interventions [33]. Despite methodological potentials and strengths, a major limitation of fMRI studies is the strong restriction on movement [5].

Functional near-infrared spectroscopy (fNIRS) is a technique that uses near-infrared radiation—which may penetrate bone and biological tissue—to study the body and brain [34]. fNIRS may be used to measure biological changes similar to fMRI ([35,36,37]), and real-time measurements taken through fMRI and fNIRS show high correspondence [35,36]. However, as fNIRS measurements are based on how light is absorbed in the tissue under investigation, this limits the depth of the brain that may be investigated to 1.5–2 cm [5,36]. fNIRS has a higher temporal resolution—and far fewer restrictions on movement—compared to fMRI [5]. A recent review of the use of fNIRS to understand the effects of listening to music, playing music, and singing on brain function highlighted the broad spectrum of using fNIRS to connect music and brain function. It was found that passive listening predominantly activated the prefrontal cortex while active singing and playing instruments engaged the inferior frontal cortex and temporoparietal junction [7]. In this context, fNIRS allows for the investigation of the effect of music on the brain in a non-invasive, unconstrained manner and in ecologically valid contexts. fNIRS has been used to study violinists performing live, as exemplified by the study of Vanzella et al. [38]. In their fNIRS study, they used hyperscanning (a technique that allows for the simultaneous recording of brain activity of different subjects [39]) of selected regions of the right hemisphere in two violinists simultaneously to study their perceived role when performing given pieces of music. We used this publication as a guide for the design of our fNIRS measurements.

**Figure 2:**
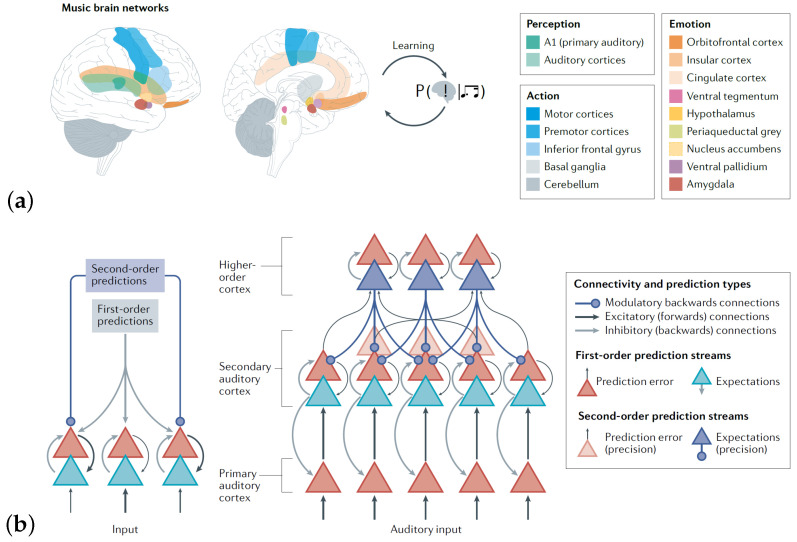
Music affects the brain through several distinct regions and accompanying networks. (**a**) An overview of how different anatomical regions are coupled to the perception of music. (**b**) The foundation of the Bayesian statistical model underlying the Predictive Coding of Music (PCM) model, which models how the brain constantly tries to predict the ongoing perceived music. (**a**,**b**) from [8]; permission for reuse of figures was granted by professor Vuust.

How music may be modeled as an active process is reflected in the Predictive Coding of Music (PCM) model [8,40]. Music engages a large part of the brain that is involved in several processes Figure 2a. PCM is an active inference model that describes how music processing can be understood as a Bayesian statistical hypothesis-driven combination of bottom-up (updating) predictions and top-down precision in prediction processes, which the brain uses to continuously predict what is most likely to happen next (Figure 2b). We were most interested in coupling changes in brain dynamics in anatomically restricted areas when musicians performed repeated repetitions; that is, we aimed to couple changes in brain activity to the context in which the professional artists were performing their art.

We therefore set out to develop a methodology whereby we could observe the act of music making in a real-time setting when the musicians are part of a symphony orchestra, and, if this was possible, how such real-time reflections could potentially relate to given subregions of the cortex and the number of repetitions of the repertoire performed.

**Figure 3:**
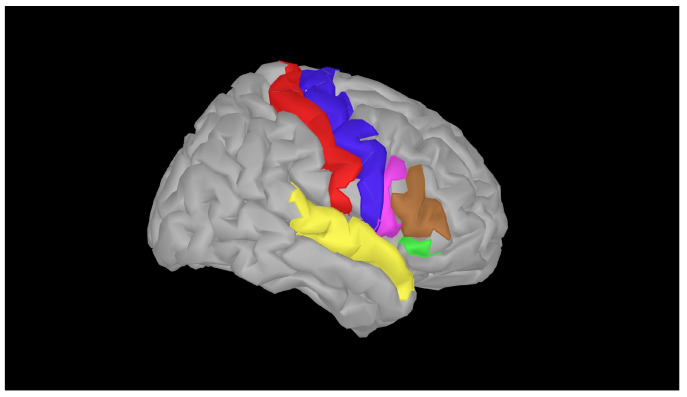
In this pilot study, we followed the selected activation of given regions of interest (ROIs) in the right hemisphere of two violinists performing in a symphony orchestra. The ROIs modeled were the postcentral gyrus (POG, red), the precentral gyrus (PRG, blue), the superior temporal gyrus (STG, yellow), and the three subcomponents of the interior frontal gyrus (IFG), pars opercularis (pink), par triangularis (brown), and pars orbitalis (green), all defined through the Destrieux atlas [46].

To investigate the ongoing change in brain activation related to music making through sequential concerts, we chose to model this by measuring the changes in the precentral gyrus, postcentral gyrus, superior temporal gyrus, and inferior frontal gyrus, abbreviated as PRG, POG, STG, and IFG, respectively (Figure 3). These subcomponents were chosen because the primary motor cortex, responsible for the control of voluntary motor movement, is located within the PRG ([41]); the primary somatosensory cortex, a significant region responsible for proprioception, is located within the POG ([42]); the ultimate target of afferent auditory information is the auditory cortex ([43]), and the STG houses the primary and secondary auditory cortices [44]. Additionally, the right IFG has been associated with attention, motor inhibition, imagery, and social cognitive processes [45].

Following advice from [38], we restricted fNIRS measurements to the right hemisphere. This was chosen to avoid the optode positioning in the fNIRS headcap potentially affecting the violinist’s performance (e.g., equipment touching the shoulder/chin rest). The regions of interest (ROIs) of the right hemisphere are shown in Figure 3.

The use of fNIRS for investigating the relationship between music and brain function is still recent, and reviews have highlighted limited focus on specific brain regions ([7]) and unexplored potentials within hyperscanning for investigating interbrain synchronization [47]. As far as the authors know, this is the first time fNIRS hyperscanning has been used to record/map a sequence of entire concerts of musicians performing in a symphony orchestra. In collaboration with SSO, we used fNIRS hyperscanning to investigate the ongoing hemodynamic changes in a first violinist and a second violinist (roles within the orchestra playing different musical arrangements of the same compositions) during seven sequential concerts.

The findings show that fNIRS can be used as a tool to answer questions about how performing music is expressed in the brain. We—somewhat arbitrarily—chose to investigate violinists due to references and cooperation with SSO, but this approach may be generalized to investigate most artistic performances, with or without musical instruments.

## 2. Materials and Methods

This project was a collaboration with SSO. SSO was founded in 1938 and is one of the most prosperous orchestras in Scandinavia. SSO rehearses in Stavanger Konserthus—ranked as one of the best acoustic halls in Europe—and is composed of 85 musicians from 22 different nations [48]. Two violinists from the orchestra volunteered and agreed to be recorded with fNIRS, and both were equipped with the Brite MKII fNIRS headset from Artinis [49].

**Figure 4:**
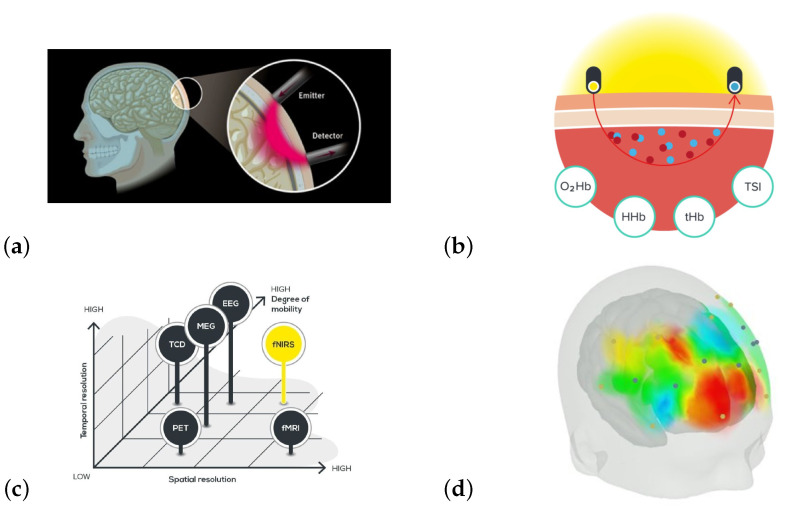
(**a**) The principles behind fNIRS; how the near-infrared light from the emitter is scattered in brain tissue—modeled through the modified Beer–Lambert law—and registered by the detector; (**b**) a schematic representation of how NIRS scattering is reflected through oxygenated and total hemoglobin, as well as the tissue saturation index (TSI); (**c**) the relative positioning of fNIRS technology with respect to spatial and temporal resolution as well as degree of mobility. The spatial resolution was sufficient for mapping the parts of the cortex that we were interested in, the temporal resolution was sufficient for the overall changes during the concerts, and the degree of mobility allowed us to set up the equipment and leave the musicians alone to perform their art; (**d**) an example of how 3D modeling in OxySoft may be performed in real time. (**a**–**d**) from ([50,51,52]), respectively; (**a**) reprinted with courtesy of ISS, (**b**–**d**) reprinted with permission from Artinis.

We used the protocol described in [38] as a guideline, following their advice to restrict the measurement to the right hemisphere so as not to interfere with the performance of the violinist. Following [38], we mapped the changes in the right motor cortex and the sensorimotor cortex and kept an area of the dorsal frontal cortex as a control region. An explanation and visualization of important fNIRS principles are provided in Figure 4.

### 2.1. Motor Paradigm, Live Concerts

This study was conducted in an exploratory and observational manner. The researchers conducting the fNIRS measurements adapted to the prearranged concert setup, and improvised if problems occurred. All that was required from the musicians was that they met the researchers in advance of the concert in order to have the fNIRS headcaps fitted and the quality of the signal tested.

Subsequently, the researchers initiated the fNIRS recording and subjectively placed synchronized events/tags in each recording for subsequent analyses. The motor paradigm, therefore, was the concert as planned by the concertmaster:

*Prelude from La Traviata* (Verdi), *Hedwig’s Theme* (Williams), *Kjempeviseslåtten* (Sæverud), *Symphony No. 8 in B minor, 1st movement* (Schubert), *The Firebird, 1st and 3rd movements* (Stravinsky), *Adagio for Strings* (Barber), *William Tell Overture* (Rossini), Excerpt, *Threnody, Morning Mood, Ingrids klage* and *Norsk Dans no. 2* (Grieg), *Gammel Jegermarsj* (Schiöldberg), work by Izabell, and a student composition.

### 2.2. fNIRS Acquisition Protocol

The musicians were fitted with Brite MKII fNIRS headcaps (Artinis), featuring a channel setup of two 2 × 4 channels and two 2 × 5 channels, where the latter includes two short channels (SSCs), Figure 1a. The use of SSCs allows for continuous measurement of the hemodynamic changes in regions of the head above the brain. As these channels also move with the optodes measuring the regions of interest (ROIs), SSCs may be used to decrease interference from both movement artifacts and the fNIRS signal from superficial parts of the head and brain (the “gold standard” for reducing responses from extracerebral tissue [53]).

Each Brite headset was controlled by separate laptops but was synchronized by setting up a Lab Streaming Layer (LSL, ref. [54]) between the laptops. One laptop/headset was chosen to be the master controller; events input on this laptop were streamed to the other recording, ensuring synchronization for later analyses.

In the control room of SSO, the technicians had access to a projector that was used to display what was shown to the audience. This projector was connected via HDMI to display the laptop screen of interest. We wanted to show the changes in two hemispheres across two different brains, but OxySoft only allows for the display of one brain at a time. Two hemispheres from different brains can be shown, but only by displaying both the left and the right hemisphere. If the experiment contains two right hemispheres, one must be chosen and mirrored to a left hemisphere. We therefore chose to use the application OBS Studio to set up a connection between the laptops in advance and project a combined display of the two hemispheres (including a picture of the violinist and the role of the violinist) to the audience during the concerts (Figure 1b).

The two violinists met with the researchers 15 min in advance of each concert.

### 2.3. fNIRS Real-Time Data Visualization

Once the recording of the fNIRS signals for both performers had been initiated, the researchers continuously monitored the ongoing recording to include labels for subsequent analyses and to ensure that the signal maintained sufficient quality throughout the concerts.

**Figure 5:**
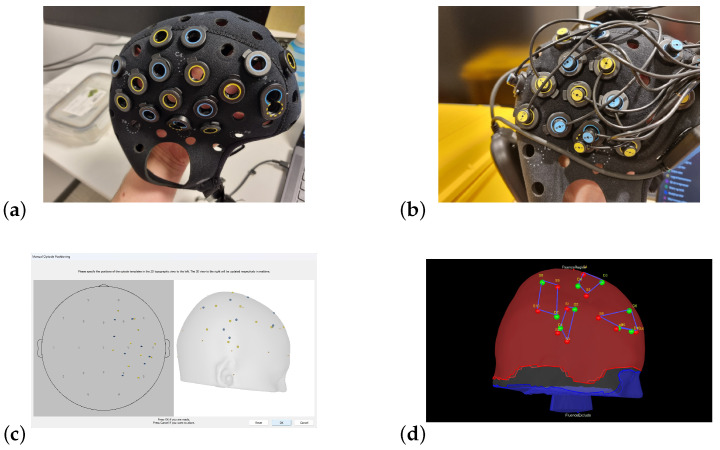
(**a**) Positioning the optode holders; (**b**) Group 1, Sensorimotor: Tx1, Rx2, Rx1 (Tx2 SSC, Group 5), Tx3; Group 2, Sensorimotor: Tx4, Rx3, Rx4, Tx5; (**c**) manually registering the coordinates of the optodes in OxySoft, visually; the input coordinates may thereafter be extracted; (**d**) the extracted OxySoft optode coordinates were later input and adjusted according to the Colin27 model in Brainstorm.

To display a changing topographical model of the cortex, a model including the coordinates for the positions of the optodes was prearranged so that the recorded signal from each channel was displayed in the corresponding areas of the cortex Figure 5a–c.

Real-time visualization of the hemodynamics of the two violinists was shown only during a subset of the concerts. When reaching the piece of the concert where the brains were to be displayed, the HDMI cable was switched from the concert technician’s laptop to the researchers’ laptop.

### 2.4. fNIRS Preprocessing

To investigate the hemodynamic changes throughout the concerts, we used OxySoft ([55]), Matlab ([56]), and the Matlab toolboxes Homer2/Homer3 ([57,58]) and Brainstorm [59]. Regarding Homer2/Homer3, standardized approaches were used, and, for Brainstorm, we used the preprocessing pipeline and parameters recommended in a workshop conducted in 2022 ([60]), apart from the high end of the bandpass filter, where we used 0.5 Hz, as in a workshop from 2018 [61].

### 2.5. Estimating Sources of Hemodynamic Change

Modeling the sources within the brain (*source space*) that potentially cause the hemodynamic changes measured by the optodes (*sensor space*) relies on a *head model*, which models the electromagnetic properties of the head and sensor array (also known as a *forward* model/solving a forward problem [62,63]), and a subsequent *inverse model* [64]. The inverse model is an estimated solution of how the brain activity of potentially thousands of locations in a source space is detected by far fewer sensors in the sensor space; an infinite number of source patterns may equally well explain the sensor data [65]. These estimations were accomplished by using Brainstorm [66].

The optode setup/experimental design did not allow for the mapping of the entire cortex, so decisions were made regarding the areas of the cortex that were of highest interest. A forward model or head model was first calculated to determine the sensitivity of the measurements. Once the sensitivity of the given regions was calculated, the recorded fNIRS signals could be used to estimate the sources within the brain through an inverse model.

We used the well-characterized adult male template brain, the Colin27 model ([67]), to create a forward/head model based on changes in the optical density of the recordings (after detecting and removing bad channels) and downloaded the corresponding fluences from a Brainstorm-related webpage ([68]). The Brainstorm translation of the coordinates of the sensor array is seen in Figure 5d.

We modeled how the experience of the violinists’ performance changed while they were performing by examining changes in HbO, HbR, and HbT concentrations in four ROIs in the right hemisphere: (1) the PRG, (2) POG, and (3) STG using vertex vectors obtained through Cat12 segmentation in Brainstorm [69]. We also investigated the change in a fourth ROI, the combined IFG; all ROIs were defined through the Destrieux atlas [46].

Three protocols were developed, each suited to answering different research questions: (1) an overall average of the development of each separate channel throughout each piece, (2) an analysis of the hemodynamic changes during the first 20 s of each piece, and (3) a model for investigating the hemodynamic changes in the middle of a subset of five longer pieces. An important difference distinguishing the first approach from the latter two is that the first only takes the performance of the violinists into account and is purely an averaging approach. The first approach neither takes any external reference into account nor corrects for baseline values. This is carried out in the two Brainstorm analyses.

#### 2.5.1. Overall Channel Average

During the concerts, events were placed in OxySoft to signal information about each segment of the recordings. The event information was contained in the Matlab-conversion from the OxySoft files (.oxy5) to the Homer3-based format (.snirf) through the oxysoft2matlab function [70].

In Homer3, we conducted the standard processing of the fNIRS recordings through the predefined Matlab stream: Intensity to Delta OD, BandpassFilter Auxiliary, BandpassFilter OpticalDensity, Delta OD to Conc., and Block Average on Concentration Data, which converts the data from raw signals to changes in optical density and yields the average change in concentration. After Homer3 preprocessing, the data were output to a column-based spreadsheet, where we wrote tailored Matlab scripts to convert and summarize the changes in total hemoglobin for each piece by each violinist.

To investigate the value of using the SSC, we tested a procedure where we subtracted the mean value of the two SSC channels from all the other channels. As this form of signal correction may (also) be used to indicate channels where the signal may be less trustworthy, we chose to set the 3D columns where the channel signal was negative after the SSC subtraction equal to zero. This method of analysis does not take any baseline into account. We used this approach—the averaged SSC subtraction—to provide an indication of the relative size of channel values versus short-channel values, measuring signals from cerebral versus extracerebral tissue, respectively [53].

#### 2.5.2. Changes, Initial Sections

**Figure 6:**
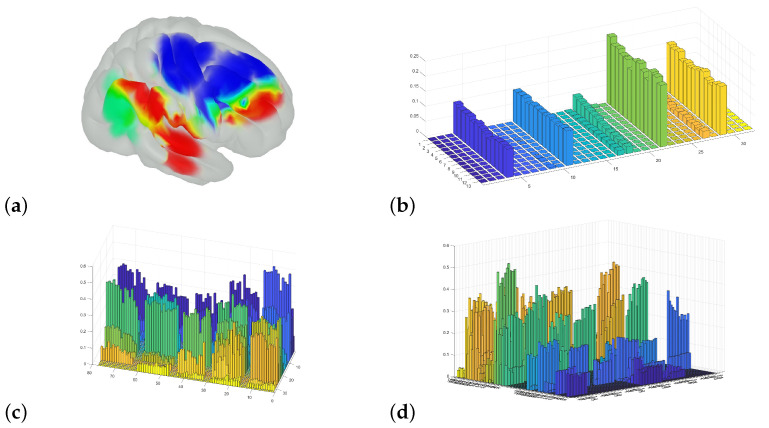
(**a**) The optode coordinates manually input in OxySoft, where the scale of the fNIRS signaling was chosen arbitrarily at each concert to maximize changing dynamics for the interest of the audience, predominantly children; (**b**) averaged and SSC-corrected Homer3 analysis of the 1st violinist performing concert 1, where channels located over potential areas of importance for the action conducted/experienced become evident; (**c**); average and SSC-corrected Homer3 analysis of the 2nd violinist performing concerts 1–7, where we encountered problems with the fNIRS-recordings of the 2nd violin in concerts 4 and 6; (**d**) overall average and SSC-corrected signal change in each channel for each of the violinists in the 7 concerts. The dimension of the z-axes in (**b**–**d**) is “Molar mm”.

For the analysis of the hemodynamic changes at the introduction of each piece, we analyzed this using the Matlab toolbox Brainstorm ([66]) with the Nirstorm plugin (fNIRS analysis, [59]). When transferring the recordings from OxySoft to Brainstorm, we chose to use the Homer2 file format (.nirs). When developing the procedure, Brainstorm workshops were used to establish the protocol ([60]) and, e.g., the coordinates for positioning of the optodes were used with a reference to these (but these coordinates were manually adjusted later on to fit our analysis). A snapshot of the OxySoft display, which was projected to the audience (where the optode positions were visually “dragged and dropped”), is shown in Figure 6a. These displayed coordinates in OxySoft were later exported to Brainstorm for the following analyses.

To compare the estimated sources from each violinist, we modeled the hemodynamic changes on the Colin27 template cortex. This also made the development of the protocol more rapid, as the fluence of the Colin27 cortex has been modeled/calculated ([60]). By using the Colin27 anatomical template and the precomputed fluences, we can model and investigate the changes in both violinists, allowing for direct comparison, despite the reduction in accuracy for each individual.

We chose the duration of the analysis to ensure a sufficiently long segment for differentiating the pieces while being short enough to include most of the performances during the concerts. We therefore selected to analyze a 5 s baseline in advance of each piece and 20 s of each piece. The Brainstorm analysis/modeling thus performs a baseline correction. The Brainstorm preprocessing included the detection and removal of bad channels, conversion from raw signal to changes in optical density, bandpass filtering, and conversion from optical density to changes in oxygenated, deoxygenated, and total hemoglobin.

#### 2.5.3. Changes, Mid Sections

To investigate the hemodynamic developments taking place during the performances, we chose to examine sections of pieces neither close to the start nor the finish. Therefore, we selected five pieces that had a duration longer than three minutes, used a baseline of 20 s before each piece, performed the same Brainstorm analysis, modeled the initial three minutes, and extracted the hemodynamic developments from 60 to 120 s through tailored Matlab scripts.

From these models, the average changes in the vertices defining the volumes of interest in the right precentral, postcentral, and superior temporal gyrus were extracted. A similar analysis of the combined three components constituting the right inferior frontal gyrus (rIFG) was also conducted.

### 2.6. Averaging and Confidence Intervals

For the procedure in “2.5.1. Overall Channel Average”, the musical pieces were first isolated in time (based on the labeling input during the fNIRS recording), and the time in seconds was translated to a cell number through the sampling frequency used in the recording (75 Hz). The Homer3-processed result matrices from the 36 channels (2 × 16 conventional channels, plus 2 short channels and 2 wavelengths) were averaged based on each segment in isolation. There were no references to a baseline in these analyses, other than the automatic fNIRS signal calibration performed in advance of the concerts, when the musicians were in a relatively relaxed state. The greatest importance for these analyses was to assess the relative scale of the fNIRS signal resulting from the conventional channels, measuring the hemodynamics in the chosen cortical areas, and the short channels, measuring extracerebral hemodynamics.

For calculating the averages in “2.5.2. Changes, initial sections” and “2.5.3. Changes, mid sections”, the baseline correction was taken into account in the modeling through Brainstorm. The calculated result matrices for the changes in total hemoglobin (HbT), oxygenated hemoglobin (HbO), and deoxygenated hemoglobin (HbR) were extracted, Brainstorm scout files were used to define the rows of interest from the 10,000 row matrix, and these vertex values were averaged in time, and also with respect to the number of vertices for the given ROI.

For calculating the 95% confidence interval (CI), we used the resulting source model matrices (X) to calculate the standard error (SEM), the statistical toolbox in Matlab to calculate the Student’s t inverse cumulative distribution function in order to obtain the T-score (T), and calculated the CI through Equation (1):

(1) CI=mean(X)+T·SEM

**Figure 7:**
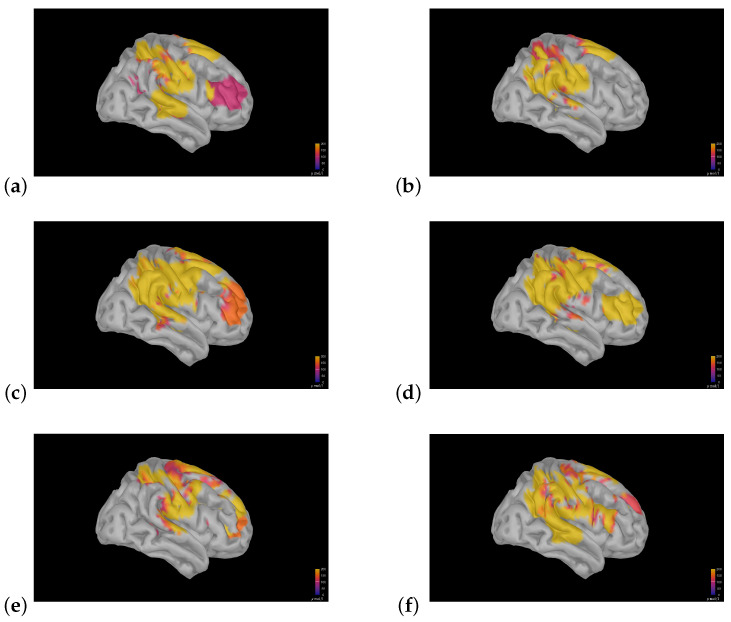
These figures display the total averaged hemodynamic change (HbT) during 60 s in the middle of 5 longer pieces of the repertoire. The models were baseline-corrected in advance of the averaging, and all averaging was conducted on a per-vertex basis. The figures display the following: (**a**,**b**), the respective changes during concert 1 and 7 for the 1st violin; (**c**,**d**), the respective changes during concert 1 and 7 for the 2nd violin; (**e**,**f**), the overall average for the given period of time during all the concerts for the 1st violin and the 2nd violin. The change in value displayed in (**a**–**f**) are *absolute*.

We also calculated an overall average for selected concerts based on the total change in oxygenated and deoxygenated hemoglobin (HbT), presented in Figure 7. This was carried out to illustrate the areas of importance covered by (and limitations within) the model based on the optode positioning during the recordings before advancing to the “functional dissection”.

## 3. Findings

This study showed that real-time visualization of ongoing hemodynamic changes in the brains of musicians in a performing symphony orchestra can be achieved. The fNIRS software OxySoft 3.5.15.2 and equipment worked well in most cases, but signal and software problems led to the fNIRS recordings from concert 4 for the first violinist and from concerts 4 and 6 for the second violinist being either lost or untrustworthy. Subsequent analyses show that this methodology may be tailored to investigate detailed changes while performing music. The overall goal of this study was the real-time visualization of brain activation, and as the researchers conducting the science did not hinder the concerts, this corroborates how fNIRS may be used to investigate how art is expressed in the brain in natural contexts. Our findings are intended to be understood as showing principles of what may later be investigated with stronger experimental control rather than as conclusive results.

For the real-time visualization, the developed protocol worked well, with an example shown in Figure 1a,b. By staging and rearranging the recording in advance, the only thing required of the violinists was that they meet the researchers before the concert, have their fNIRS caps fitted, and have the fNIRS signal checked (which took just a couple of minutes), allowing the fNIRS data to be streamed live thereafter.

It was only one segment where the brain activity of the violinists was to be displayed. The researchers tested the scaling of the color coding for the brain activity in advance of this piece to yield a range where the output from the fNIRS recording created the most interesting visual changes for the audience to observe. These concerts were arranged for an audience predominantly consisting of children, which heightened the importance of displaying a vivid brain model (Figure 6a). The researchers were well aware that altering the topographical live model did not interfere with the fNIRS raw data.

For the development of the Matlab analysis of the Homer3-processed results, we used the standard default processing steps suggested by Homer3. After the Homer3 processing, we imported the results from Homer3 into a matrix in Matlab, and used the recording frequency (75 Hz) to translate the event markers to cell numbers. From these cells, we calculated the average fNIRS response for each of the channels for each piece from both violinists. From the resulting calculation for each piece, we averaged the short-channel signal for that piece and subtracted this value from the other channels. Thus, we used the SSC for its intended purpose: to correct artifacts resulting from movement and superficial hemodynamic changes.

The results from the SSC subtraction of the first violinist (e.g., for the first concert, Figure 6b, and all the concerts, Figure 6d) show that the SSC signal was relatively high compared to the recorded signal in the other channels, and that this difference was smaller for the second violinist (Figure 6c).

**Figure 8:**
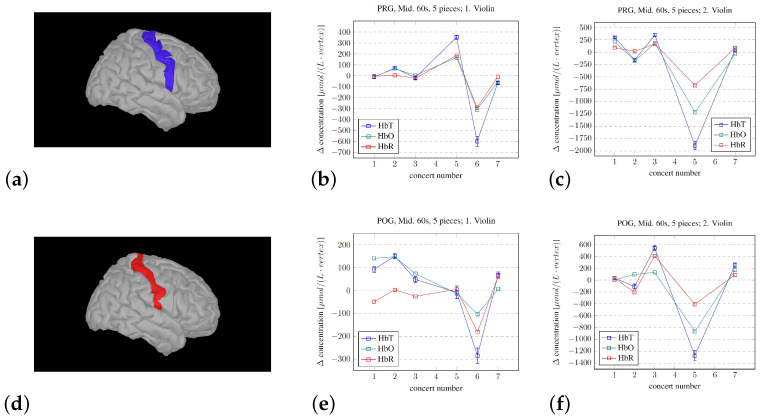

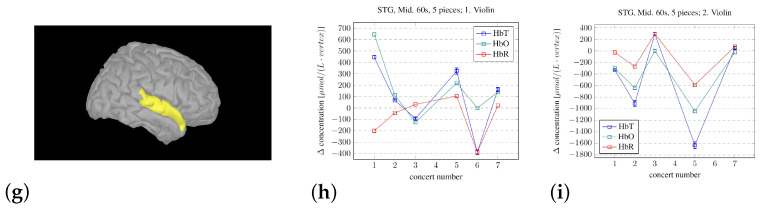
The ROIs—PRG: (**a**), POG: (**d**), STG: (**g**)—along with the hemodynamic response recorded from 60 s to 120 s in pieces lasting longer than 180 s (**b**,**c**,**e**,**f**,**h**,**i**). This period of time was chosen to investigate a period neither close to the start nor close the end of the pieces in question. The calculation of the sources was conducted through Brainstorm and custom Matlab scripts. The y-axis dimension in (**b**,**c**,**e**,**f**,**h**,**i**) was chosen as Brainstorm yielded the change in HbT concentration; this was averaged in time, summed over the vertices of the ROI in question, and multiplied with the reciprocal of the number of vertices in the ROI. #vertices, 10,000 vertex head model: PRG: 107, POG: 97, STG: 92.

**Figure 9:**
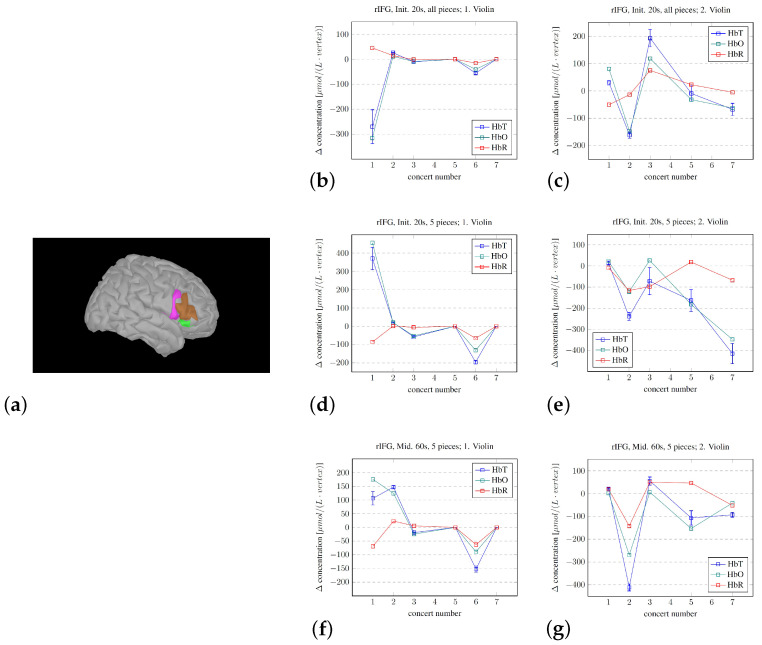
The development of the activity in the right inferior frontal gyrus (rIFG) for both violinists throughout the different concerts. (**a**) The rIFG as defined by through the Destrieux atlas, through the 3 components: pars triangularis, pars opercularis, and pars orbitalis. The developing hemodynamic response analyzed through the initial 20 s of all the pieces (**b**,**c**), the hemodynamic response during the initial 20 s of the 5 selected longer pieces (**d**,**e**), and the hemodynamic response during 60 s in the middle of the selected 5 longer pieces (**f**,**g**). An explanation of the y-axis dimension is given in the caption of Figure 8; the summed number of vertices in the 3 subcomponents of the rIFG in this 10,000 vertex head model is 144.

The use of Brainstorm allowed for investigating and modeling the hemodynamic changes in more detail. An investigation of averages over the entire measured area is shown in Figure 7. The selective changes in the three ROIs—pre- and postcentral gyri and the STG—are shown in Figure 8, and the changes in the summed rIFG under three different experimental conditions are shown in Figure 9. The analysis of the overall averages implied—corresponding to the Homer3 analysis in Figure 6—that the signal intensity of the first violinist was generally lower than that of the second violinist. The detailed investigations of the ROIs in Figure 8 and Figure 9 show that such analyses can be conducted, but correctly imply that the limitations in the spatial precision of fNIRS investigations (∼1 cm) must be taken into account during the experimental design (e.g., similarity of/corresponding changes in the PRG and POG).

## 4. Discussion

In this study, we set out to map the experience of performing with a symphony orchestra and how this may change through repeated concerts. We developed protocols for using fNIRS to observe real-time changes in brain activity and methods to quantify these changes. We discovered that the fNIRS methodology is an effective way to passively observe any changes that might be related to how the musicians experience their own performance. The importance of the good cooperation and willingness of musicians in SSO throughout this project cannot be overstated and is greatly valued.

As this was the first time the violinists were exposed to fNIRS, after having tested the equipment once during the dress rehearsal, the first violinist said that the cap was a bit tight and that he experienced a slight headache. As the optode holders offer two positions concerning distance to the head, the researchers positioned all the optodes in the outermost positions in the holders. This led to the resolution of the problems experienced by the first violinist. The second violinist did not experience any similar problems. This was the first extensive pilot study using the fNIRS equipment, and since the second violinist did not experience similar problems, less focus was therefore set aside to investigate this. Judging from the findings exemplified in Figure 6, the signal quality—on a general level—seemed to be sufficient for the recording of the second violinist, and perhaps to a greater extent compared to the first violinist.

The OxySoft software includes a signal quality check for all the channels from all the optodes before initiating a new fNIRS recording, and the researchers verified that the signal quality was sufficient in all channels prior to starting the recordings of all the concerts. That being said, the optode depth may have been heterogeneous with respect to the second violinist. The noted OxySoft quality check includes automatic scaling and thresholding of the signal to homogenize the channels, which should have adjusted or reduced the signal for the second violinist if some channels—with optodes potentially in the deeper position—were excessively high. A common problem with fNIRS recordings is that the amount of signal depends on the amount of hair (and the level of pigmentation of the skin) of the person being investigated. The first violinists did have more hair than the second violinist, which may also explain why the overall signal level was higher for the second violinist than for the first, as implied in Figure 7. (We also conducted fNIRS recordings of elderly volunteers in another pilot study, some of whom had an extensive amount of hair, but hair that was completely white. Since this did not interfere with the fNIRS signal, this verified that it is how the hai -pigmentation influences the scattering dynamics of the light which is of importance, not the hair per se.) For future research, we recommend that the optode depth be excluded as a parameter.

What also became evident, in retrospect (exemplified in Figure 6d), is that the magnitude of the SSC for the fNIRS recordings of the first violinist may have resulted in a significant portion of the fNIRS recording for this violinist being predominantly the result of noise.

Using OxySoft was experienced as easy and intuitive by the researchers, and the software itself, along with the Bluetooth connection to the fNIRS equipment on stage, was stable and trustworthy in most cases. However, we nevertheless experienced some software and connection problems. One of these instances occurred during the dress rehearsal when one of the violinists suddenly moved outside the Bluetooth range by leaving the concert stage. This was due to insufficient information being supplied by the researchers. After this information was provided, this problem did not reoccur. We did experience the loss of the fNIRS signal from the violinists for unknown reasons, and we also encountered a problem related to entering or defining events in OxySoft. However, these issues were the exception rather than the norm. When the software froze and we were forced to restart it—in the middle of a concert—combining the subcomponents in an effort to generate a complete whole of the entire concert proved to be difficult for several reasons. For example, the signal quality check/normalization was conducted during a concert without the violinists’ awareness, which means that the signal was by no means initiated in a resting state. Not having the same signal reference point—since this is automatically tuned by the software—makes it difficult to compare or translate the relative signal intensities in terms of what they may imply. It also became more difficult to compare the subcomponents within the concert, as manual and subjective stitching of the results became mandatory. The exportation of processed results to Matlab and combining the subsegments from concerts where this was experienced was tested; however, it became evident that numbers extracted from different subsegments did not imply the same hemodynamic change. Not being able to show two brains in a hyperscanning protocol, where the two laptops and recordings were synchronized through an LSL, was experienced as a flaw; however, the OBS Studio solution resolved this problem. Overall, the Artinis equipment and software met our expectations.

Brainstorm was experienced as easy to implement, and the way that this software opened up for “functional anatomical dissections” made it possible to investigate changes in the brain from a new perspective. For the investigations of the PRG, POG, and STG, our findings show a spread around zero. However, regarding the missing data points for the fourth concert for the first violinist and the fourth and sixth concerts for the second violinist, this may have resulted in findings from these regions that are easier to interpret. However, we must also consider how methodological limitations influence the models in use. Comparing how, for example, the superior temporal gyrus is defined in the Destrieux atlas (and its corresponding vertices in Brainstorm, Figure 8g) with the real-time plotting of brain activity measured during the concerts (Figure 6a), it is evident that the positioning of the optodes is not sufficient for a thorough mapping of the developing activity of the STG. That being said, since this was an exploratory pilot study aimed more at replicating a published approach than at conducting precise mapping for anatomical inferences, it did serve its purpose in demonstrating the potential. This topic is further discussed in the limitations. This procedure opens up opportunities to investigate how the degree of activity in selected regions of the brain may change over time, and this perspective could be highly valuable for exploring topics such as learning to play an instrument or learning in general.

The findings from investigating the developing activity in the (right) IFG (rIFG) share similar limitations to those discussed earlier regarding signal quality, but the Destrieux atlas definitions imply that the three subcomponents of the rIFG are covered by the channels. It is interesting to note that, regardless of how we choose to analyze the subcomponents of the concerts, the activity in the rIFG does seem—after a larger variation in the earlier concerts—to tend to decrease toward zero. This is important considering the potential role of the rIFG in attention, as fMRI reviews of the use of methylphenidate to enhance concentration in the treatment of attention-deficit/hyperactivity disorder (ADHD) show how this prescription stimulates activation in the rIFG ([71,72]). Investigating the effects of methylphenidate has also been carried out with fNIRS, which corroborates the fMRI results ([73]), but the fNIRS literature on this topic is still limited [21]. Targeted training of the rIFG through real-time fMRI neurofeedback did show promising results for the treatment of ADHD ([74]), but the authors were not able to replicate their results on a later basis [75]. The training of attention has also been tested through real-time fNIRS neurofeedback, where the channels selected for feedback were tuned to each participant [76]. Our group has also developed real-time fMRI neurofeedback ([77]) and real-time fNIRS neurofeedback (unpublished), and the use of these procedures to investigate changes over time through both technologies may provide an important perspective. The correspondence between fMRI and fNIRS is high (e.g., [35,78]), implying the potential for combining the strengths of each technology.

The noted pilot studies on elderly volunteers inspired further investigations into this population, which may be especially promising regarding the use of fNIRS to study, for example, the development of Alzheimer’s disease (AD). A review of the use of music therapy for treating Alzheimer’s disease (AD) argued that music interventions may potentially delay and decelerate the neurodegeneration of individuals at risk for AD [79]. However, a 12-month randomized pilot study showed that this approach was not feasible for individuals with mild to moderate AD, indicating that music therapy should be implemented at an earlier stage [80]. We used the same fNIRS equipment and protocols to test both the violinists and healthy elderly volunteers in a music therapy pilot study, and none of the participants complained, neither about the fNIRS testing nor the music therapy. We therefore corroborate [79] in suggesting the use of music therapy for the elderly and recommend conducting the measurements through fNIRS.

In this project, we were primarily interested in making the real-time fNIRS measurements of the violinists work. As this was our main interest, we did not perform sufficient qualitative mapping of the experience, nor did we arrange for an adequate experimental control. We recommend this as a natural follow-up to our study. Performing additional qualitative mapping of the experience, such as how the musicians felt during the concerts, how they felt when performing each piece, and whether this feeling changed as a consequence of the difficulty level of each piece, may allow for a triangulation of the qualitative and quantitative data. This could help to specify, e.g., how the subjective experience may be coupled to specific regions of the brain, and how this may change over time if repeated measurements are conducted. The lack of a thorough experimental control casts doubt on the validity of using our findings for anything more than as a source of inspiration for follow-up experiments.

An important point or limitation one always needs to consider when using fNIRS and fMRI is that these techniques do not reflect brain activation per se, but rather an overarching change in oxygenation levels that reflects a metabolic change [81]. The temporal dimensions of neuronal activation are in the range of ms, but the strongest increase in BOLD signal amplitude occurs 4–6 s after neuronal activation [81]. We investigated these hemodynamic changes during pieces by averaging over time windows exceeding this threshold; however, to enhance the temporal precision of the investigations, additional sensors may be incorporated. A recent review highlighted how wearable technologies in the last decades—e.g., a Musicjacket, stress-related physiological parameters, foot gesture recognition, real-time feedback, machine learning, and the Internet of Things (IoT)—have been used to study musical experience [14]. Using such additional sensors may both decrease the limitations regarding temporal resolution in our study and increase the dimensions of how the musical experience may be understood.

The use of fNIRS to investigate performing musicians can also be extended to explore different instruments (e.g., how guitar improvisation may activate ROIs coupled to spontaneous creativity [82]); hyperscanning—as used in this study—may investigate the interactions of musicians ([47]) and may potentially provide insights into what is required for an “instrument to be an instrument” [3]. Musical instruments do not need to fit a predetermined, conventional stereotype. The important aspect is how interacting with the musical instrument affects one’s experience and thinking; thus, “air instruments” may also induce such changes [3]. The use of fNIRS could shed light on how interacting with different instruments affects different parts of the brain; if selected parts of the brain nevertheless show corresponding activity changes regardless of the instrument, these regions could be important for the essence of the experience of “performing music”. This may be further extended to investigate how the state of mind of musicians may change while performing to, e.g., differentiate and quantify a “wandering mind” from a “surfing mind” [83].

As for the four questions we set out to answer, as stated in the Introduction, the following is noted:

1. We were able to develop a protocol for displaying changes in the brain—in real time—that reflect the experience of performing the violin in a symphony orchestra.
2. The development of the procedure for averaging the hemodynamic changes lasting the longevity of the pieces did show systematic changes. The observed difference between the musicians in this pilot study may corroborate the results from [38], where they found that the violinists experienced leader and follower roles while playing first and second violin, respectively. But, regardless of the roles, the activity in the measured parts of the right hemisphere seemed to decrease. But, we cannot disregard the influence/disturbance by hair. The musician with the most hair did have a very high average signal for the SSC, which, for most channels, became the dominant signal.
3. The Brainstorm modeling estimating the fNIRS signal sources does indicate a relative activity change of the subcomponents throughout the concert sequence. Such modeling may be used to identify the brain regions that are most active or important for the overall experience.
4. At least on a “proof of principle” basis, there are several limitations in our approach, but we showed that the procedures that we developed may be used to track the developing experience of professional musicians. However, the mere fact that the volunteers were indeed professionals likely reduced both the change in and the variability of the measured fNIRS signals during the concerts in the chosen regions of the right hemisphere. If we assume that attention is required for updating hypotheses, the decrease in the variability of the activity level of the rIFG throughout the concerts implies that a decreasing level of attention was needed over time. This again implies that the hypotheses became better through practice/exposure, which is in line with the PCM model.

## 5. Limitations

First and foremost, this study was conducted in an exploratory manner. We did not have all the procedures in place in advance of starting the testing. This led to, for example, not informing the violinists about the distance limitations in the Bluetooth connection, and also not positioning the optodes to fully cover areas such as the superior temporal gyrus. For researchers aiming to fully and thoroughly exploit the potential of how Brainstorm may be used for functional anatomical dissection, we advise planning the optode locations according to how the ROIs are defined in brain atlases within Brainstorm. We also recommend conducting pilot studies to test the activity in any chosen ROI(s) and to verify the positioning of the channels selected to measure them.

When the researchers placed the events/labels during the fNIRS recordings, dictating the subsequent analyses, this was performed by close observation of the conductor and orchestra. However, despite there being three researchers evaluating the conductor/a live performance (and potentially because of it being three people considering this), the manual placement of the labels does include some errors. And, the fact that there were positions where numerous labels had to be put in due to uncertainty/misunderstandings of the performance and that all these labels had to be reinterpreted during the analysis process also contributes to reducing the precision of the labels and the findings. The accuracy of these labels is very important as it determines the thresholds/cell and column numbers on which the analyses are defined. This is true for the overall Matlab SSC correction in Figure 6b–d, but also for all the Brainstorm analyses, especially the analyses in Figure 8 and Figure 9, as these labels define the important points in time that separates the task period from the preceding baseline/rest period used for calculating the models. We recommend that the conductor is given a tool to unambiguously define these points in time for later replications.

How the automatic calibration of the fNIRS signal quality affected the subsequent recordings may also be discussed. This calibration sets the standard for the signal intensity for the rest of the recording. This was performed in advance of the concerts. It might be that the musicians were slightly anxious in advance of the earlier concerts but became more relaxed through increased repetition of the concerts. This is another dimension that could have affected the results, but as we did not gather qualitative data from the violinists, we cannot explore this dimension/subtract this effect. This is something that we recommend to be taken into account in later research, but as these were professional musicians, this effect is suspected to be of minor importance.

Of higher importance is the limited use of the information contained within the SSC measurements. This is another point that we recommend following researchers to invest more time in harnessing. The significance of the extracerebral fNIRS signal recorded by the SSCs is evident from the “make-shift” Matlab subtraction analysis of the first violinist relative to the second violinist seen in Figure 6b–d. The SSCs were not specifically labeled as this through the Brainstorm analyses.

Another limitation lies in the spatial precision of the fNIRS setup used in this pilot study. The spatial resolution of fNIRS is in the order of 2–3 cm ([84]), and this calls into question the applicability of this method in, for example, separating the activation within the PRG from that of the POG. The limitation inherent in fNIRS spatial resolution again highlights the importance of precise optode positioning, as indicated by the similarity in the changes measured in the PRG and POG in Figure 8. Changes based on measurements of the neighboring gyri modeled through Brainstorm do, nevertheless, reveal subtle differences; however, steps may need to be taken if detailed differences between neighboring areas are to be mapped, such as enhancing the density of optodes positioned over the ROI.

Another limitation—which is also a consequence of this being an exploration of a potential approach—is the lack of an experimental control in our setup. The study we chose to replicate for the positioning of the optodes when measuring violinists ([38]) did choose to map the dorsal area of the frontal cortex as a control, and, from the example shown in Figure 6a, the dorsal frontal area seems anticorrelated with the rest of the brain activity at that point in time. For later follow-up studies, we recommend increasing the experimental control. But, the degree of experimental control that can be imposed on a performing symphony orchestra without losing too much of the essence of what one aims to measure can be discussed.

Another limitation of the findings is that we chose to disregard the final recommended fNIRS preprocessing step due to a warning that we received in Brainstorm, which stated that this step led to the matrices closing in on becoming singular. To keep the data processing homogeneous and avoid warnings about model validity, we therefore did not conduct the final step, “Remove Superficial Noise”. We did conduct the detection and removal of bad channels, as well as the bandpass filtering (both implemented without any warnings). However, avoiding this last step raises the possibility that much of the signal reported in our analyses may be a consequence of superficial noise. This occurred in 3 of the 11 analyses of the fNIRS datasets, all involving the first violinist, who had the most hair of the two.

As this publication also included the testing and development of the Brainstorm analysis algorithm, the use of the precalculated fluences introduces another limitation. The precalculated fluences of the Colin27 template were only calculated for one wavelength, specifically for 685 nm, and they were not calculated for all 10,000 vertices. We used the Brite MK II with the wavelengths 762 nm and 842 nm for detecting deoxygenated and oxygenated hemoglobin, respectively. When conducting the Brainstorm analysis, a warning/note of information was displayed specifying that the fluences were not available for these given wavelengths, but that an approximation was therefore conducted. For vertices where the fluence was not precalculated, the fluence of the physically closest vertex was chosen as an approximation. This approach works for a preliminary pilot study such as this, but, for follow-up experiments, we recommend that all fluences are calculated specifically for the individual in question.

We did not have a Polhemus Digitization Stylus for the accurate definition of the optode coordinates in OxySoft. These coordinates were based on manual “drag and drop” positioning, comparing the digital fNIRS head model to the physical fNIRS head cap. The positioning of the optodes in 3D space in OxySoft—and the scaling of the coloring for the real-time data visualization—does not influence the raw data. However, the concluding optode 3D coordinates, after the conversion from 3D space in OxySoft to 3D space in Brainstorm, does affect the modeling, analyses, and results. Thus, this source of error is potentially exacerbated by the translation from OxySoft coordinates to Brainstorm coordinates. This translation was again performed manually.

## 6. Conclusions

This study has demonstrated that it is possible to quantify the evolving experience in performing musicians. There are limitations in our findings, but the mere fact that two professional violinists were successfully measured with fNIRS during seven sequential concerts as part of SSO, and that we were able to extract data representing the ongoing hemodynamic changes in the brain, is an important result in itself. This study yielded answers to all the questions posed in the introduction: we were able to show how the activity in subsections of the brain changed throughout the concerts and described how, for example, developments in the activity of the rIFG may imply that the general level of attention required by the violinists decreased as a function of the concert number. The limited experimental control affects the validity of the findings; however, since we aimed to measure performing musicians in a symphony orchestra in as natural a context as possible, a strong experimental control was sacrificed. Our findings indicate how our procedure may be used for detailed mapping of performing musicians in subsequent studies.

## Data Availability

Data are contained within the article.
